# Four years’ follow-up changes of physical activity and sedentary time in women undergoing roux-en-Y gastric bypass surgery and appurtenant children

**DOI:** 10.1186/s12893-017-0318-7

**Published:** 2017-12-11

**Authors:** Fanny Sellberg, Mikaela Willmer, Per Tynelius, Daniel Berglind

**Affiliations:** 1 Department of Public Health Sciences, Karolinska Institutet, K9, Social Medicin, SE-171 77 Stockholm, Sweden; 2 Department of Health and Caring Sciences, University of Gävle, 801 76 Gävle, Sweden; 3 Centre for Epidemiology and Community Medicine, Stockholm County Council, Box 45436, 104 31 Stockholm, Sweden

**Keywords:** Physical activity, Bariatric surgery, Children, Roux-en-Y Gastic bypass, Longitudinal

## Abstract

**Background:**

Objectively measured levels of physical activity (PA) in patients undergoing Roux-en-Y Gastric Bypass (RYGB) surgery remain essentially unchanged from before to one year after surgery. Effects from RYGB on objectively measured levels of PA among women undergoing RYGB and appurtenant children beyond one year post-surgery are unknown.

The aim of the present study was to objectively assess longitudinal changes in PA and sedentary time (ST), among women undergoing RYGB and appurtenant children, from three months before to nine and 48 months after maternal surgery.

**Methods:**

Thirty women undergoing RYGB and 40 children provided anthropometric measures during home visits and valid accelerometer assessed (Actigraph GT3X+) PA data, three months before and nine and 48 months after maternal RYGB surgery.

**Results:**

Women undergoing RYGB decreased time spent in moderate to vigorous PA (MVPA) with 2.0 min/day (*p* = 0.65) and increased ST with 14.4 min/day (*p* = 0.35), whereas their children decreased time spent in MVPA with 13.2 min/day (*p* = 0.04) and increased ST with 110.5 min/day (*p* < 0.001), from three months before to 48 months after maternal surgery. Twenty, 27 and 33% of women, and 60, 68 and 35% of children reached current PA guidelines three months before and nine and 48 months after maternal RYGB, respectively.

**Conclusions:**

Objectively measured PA in women remains unchanged, while appurtenant children decrease time spent in MVPA and increase ST, from three months before through nine and 48 months after maternal RYGB. The majority of both women undergoing RYGB and children are insufficiently active 48 months after maternal RYGB.

## Background

Physical activity (PA) provides numerous physiologic and psychological health benefits for both adults [1] and children [2]. In addition there is strong evidence indicating that sufficient amounts of moderate to vigorous PA (MVPA) [3] and to some extent also reduced sedentary time (ST) [4] are associated with several positive health outcomes as well as reduced morbidity and increased longevity.

Higher levels of PA after bariatric surgery have been associated with additional weight loss [5] and improvements in physical function [6]. There is currently limited data on objectively measured levels of PA and ST before and after bariatric surgery, especially with longer than one-year follow-up. The most recent systematic review and meta-analysis on PA before and after bariatric surgery concluded that self-reported PA increase substantially, whereas objectively accelerometer measured PA only show modest or no increase in PA, from before to one year after bariatric surgery [7]. Furthermore, approximately 90% of patients undergoing bariatric surgery are not sufficiently active one year after surgery, that is they are not meeting the current PA guidelines, for the general adult population, of more than 150 min of MVPA in 10-min bouts per week [8]. The to date only available study assessing PA, measured objectively with a pedometer, beyond one year after bariatric surgery, with three years’ follow-up, shows that levels of PA increase slightly during the first-year post-surgery, and are maintained through three years.

Roux-en-Y Gastric Bypass surgery (RYGB) accommodates drastic lifestyle changes after surgery, which may be transferred across family members. A small study by Woodard et al. suggests that if one adult family member undergoes bariatric surgery and alters his or her lifestyle behaviors, e.g., increased levels of PA, other family members may adopt similar behaviors [9]. Woodard et al. showed an increase in self-reported PA and a decrease in time spent sedentary, measured as self-reported screen-time, from before to one year after bariatric surgery. However, objectively assessed levels of PA and ST do not confirm a positive halo effect on other family members from maternal RYGB [10].

The aims of the present study are to: (i) objectively assess levels of PA and ST, among women undergoing RYGB and appurtenant children, from three months before to nine and 48 months after surgery, and (ii) to assess the prevalence of women and children meeting the current PA guidelines three months before and nine and 48 months after maternal surgery.

## Methods

### Participants

Women having at least one child between seven and 14 years of age were recruited from RYGB waiting lists (primary surgery) at five Swedish hospitals (Ersta Hospital, Uppsala University Hospital, Danderyd University Hospital, St:Göran Hospital and Örebro Hospital). In total, 69 women and 95 children were included in the study population at baseline. All women underwent laparoscopic RYGB surgery between June 2012 and January 2013. Visits to each of the participants’ homes were made by researchers three months before, nine and 48 months after surgery. All the participating hospitals obtained approval from the Stockholm Regional Ethical Review Board (no. 2009/1472–31/3), and all study participants provided written consent.

### Physical activity assessment

Levels of PA and ST were measured at all three time points with the GT3X+ monitor (ActiGraph, Pensacola, USA), which has been shown to assess PA and ST in adults with accuracy [11]. We analyzed three dimensional vector magnitude (V_m_) activity counts, recorded in 10-s epochs and then aggregated to counts per minutes (cpm), calculated as the square root of the sum of the counts on three axes.

Fifty-six women (81%) and 75 (80%) children wore the accelerometer at the three months before and nine months post-surgery measurements. The study population in the present study included 30 (43%) women and 40 (42%) children with valid accelerometer data at all three measure points. Participants were asked to wear the monitor on their right hip during all waken hours for seven consecutive days. The number of MVPA minutes in bouts of 10 min or longer (for women) were computed by an algorithm developed by Choi et al. [12], while non-wear time was defined as 60 consecutive minutes with no counts, allowing for two-minute interruptions with non-zero counts [13]. Bouts and wear-time were computed using the “*PhysicalActivity”* and “*Accelerometry”* R-packages (https://cran.r-project.org). All participants with more than ten hours of monitor wear time per day for four or more days, including one weekend day [14], at all three measure points were included in the analyses. Cut-offs to classify PA intensity were based on validation studies analyzing the V_m_ axes by Santos-Lozano et al. [15] and Hanggi et al. [16]. ST was operationalized as any minute showing less than 100 counts per minute (cpm) in adults, and less than 180 cpm in children. Light physical activity (LPA) in children was defined as 180–3360 cpm, and MVPA as more than 3360 cpm. In adults, LPA was defined as 100–3208 cpm, and MVPA as 3208 or more cpm.

### Anthropometrics

Anthropometric measures were taken for both women and children in the participants’ homes using calibrated research equipment and standardized protocols (SECA® scales and stadiometers). Body mass index (BMI) was calculated as weight in kilograms divided by height in meters squared. Overweight and obesity among children were derived by age and sex specific standardized methods proposed by Cole et al. [17].

### Surgery

Laparoscopic RYGB surgery is standardized in Sweden, hence conducted comparably at the five participating surgical centres [18].

### Statistical analysis

Means and standard deviations were calculated for anthropometric and PA behaviors for each of the three measurement points. Absolut numbers and percentages were calculated for sex, overweight and obesity in children. To test for changes between measurements we used paired t-tests to account for within-subject correlations. By design, comparisons between measurement points are also controlled for fixed factors remaining constant (both genetic and environmental factors) from pre- to nine and 48 months post-surgery (Tables 2 and 3). All statistical analyses were conducted using Stata 14.1 (StataCorp).

## Results

### Sensitivity analyses

Descriptive and anthropometrical variables did not differ significantly between women (*n* = 69) or and children (*n* = 95) included in the original study population, versus women (*n* = 30) and children (*n* = 40) included in the present study who provided valid PA data at all three measure points (Table 1). Further, we ran all analyses presented in Tables 2 and 3 including women (*n* = 19) and children (*n* = 19) who wore the accelerometer for more than five days and more than 12 h of wear time per day. However, the results did not differ noteworthy to those presented (data not shown).

**Table 1:** Descriptive characteristics of women and children, three months before and nine and 48 months after maternal Roux-en-Y Gastric Bypass surgery

Women	Pre-surgery, data on 1st measurement (*N* = 69) Mean (SD) or *N* (%)	Pre-surgery, data on all 3 measurements (*N* = 30) Mean (SD) or *N* (%)	9 months post-surgery (*N* = 30) Mean (SD) or *N* (%)	48 months post-surgery (*N* = 30) Mean (SD) or *N* (%)
Age (years)	38.8 (5.5)	39.5 (6.2)	40.5 (6.3)	43.1 (6.2)
Weight (kg)	107.4 (12.7)	108.3 (13.9)	74.5 (10.7)	73.7 (11.2)
BMI (kg/m^2^)	39.2 (3.3)	39.2 (3.4)	27.1 (3.1)	26.9 (3.3)
Waist (cm)	117.9 (9.7)	117.2 (10.8)	87.3 (7.4)	88.5 (10.1)
Children	*N* = 95	*N* = 40	*N* = 40	*N* = 40
Sex (girls)	48 (51%)	21 (53%)	–	–
Age (years)	9.9 (2.0)	10.0 (2.2)	11.0 (2.1)	13.7 (2.0)
Overweight and obesity	50 (53%)	24 (60%)	18 (45%)	22 (55%)
Obesity	16 (17%)	4 (10%)	4 (10%)	8 (20%)

**Table 2:** Accelerometer assessed physical activity and sedetary time, three months before and nine and 48 months after Roux-en-Y Gastric Bypass surgery. Differences within women tested with pairwise t-test

Women PA (*N* = 30)	Pre-surgery, 1st Mean (SD)	9 months post-surgery, 2nd Mean (SD)	48 months post-surgery, 3rd Mean (SD)	Difference 1st and 2nd (*P*-value)	Difference 2nd and 3rd (*P*-value)	Difference 1st and 3rd (*P*-value)
Wear time hours/day	14.7 (1.2)	14.7 (1.5)	14.8 (1.3)	0.04 (*p* = 0.863)	0.09 (*p* = 0.650)	0.13 (*p* = 0.524)
Counts per min/day	681.7 (233.1)	671.4 (234.6)	657.6 (235.8)	−10.21 (*p* = 0.789)	−13.88 (*p* = 0.755)	−24.09 (*p* = 0.541)
MVPA min/day	34.5 (21.5)	36.0 (28.1)	32.5 (21.7)	1.57 (*p* = 0.744)	−3.58 (*p* = 0.527)	−2.01 (*p* = 0.650)
LPA min/day	427.6 (100.1)	419.8 (90.1)	423.2 (99.2)	−7.71 (*p* = 0.603)	3.41 (*p* = 0.816)	−4.31 (*p* = 0.758)
Sedentary min/day	419.6 (115.9)	428.3 (96.5)	434.0 (114.6)	8.68 (*p* = 0.573)	5.67 (*p* = 0.702)	14.35 (*p* = 0.349)

**Table 3:** Accelerometer assessed physical activity and sedetary time in children, three months before and nine and 48 months after maternal Roux-en-Y Gastric Bypass surgery. Differences within women tested with pairwise t-test

Children PA Min/day (*N* = 40)	Pre-surgery, 1st Mean (SD)	9 months post-surgery, 2nd Mean (SD)	48 months post-surgery, 3rd Mean (SD)	Difference 1st and 2nd (*P*-value)	Difference 2nd and 3rd (*P*-value)	Difference 1st and 3rd (*P*-value)
Wear time hours/day	13.8 (1.1)	14.0 (1.4)	14.0 (1.2)	0.31 (*p* = 0.189)	−0.05 (*p* = 0.793)	0.25 (*p* = 0.317)
Counts per min/day	1133.6 (347.1)	1024.0 (334.5)	875.9 (324.0)	−109.62 (*p* = 0.032)	−148.03 (*p* = 0.002)	−257.65 (*p* < 0.001)
MVPA min/day	72.6 (37.9)	69.1 (33.4)	59.4 (35.7)	−3.51 (*p* = 0.557)	−9.69 (*p* = 0.064)	−13.20 (*p* = 0.041)
LPA min/day	446.2 (90.3)	417.9 (92.7)	364.0 (86.2)	−28.37 (*p* = 0.009)	−53.81 (*p* < 0.001)	−82.18 (*p* < 0.001)
Sedentary min/day	308.8 (113.4)	359.0 (129.9)	419.3 (113.0)	50.25 (*p* = 0.001)	60.22 (*p* < 0.001)	110.46 (*p* < 0.001)

### Descriptive characteristics

Descriptive characteristics of women and children with valid data on all three measure points (and the original study sample *N* = 69) are presented in Table 1. The mean time interval between the pre- and nine and 48 months’ post-surgery measures were 12.1 (SD = 2.4) and 42.7 (SD = 4.6) months, respectively. None of the women smoked or reported having type 2 diabetes at any of the three measure points. The mean pre-surgery age was 39.7 (SD = 6.3) years for women and 10.0 (SD = 2.2) years for children, age range for children pre-surgery was 6.7–14.0 years. Fifty-three percent (*n* = 21) of the children participating at all three measure points were girls and there were no sex differences with regard to descriptive characteristics (data not shown). There were no significant differences in the prevalence of overweight or obesity in children between pre- and nine months or 48 months post-maternal RYGB except from overweight and obesity pre-surgery compared to nine months after, 24 children vs. 18 (*p* = 0.025).

### Differences in physical activity and sedentary time

Mean numbers of days the accelerometer was worn for more than ten hours per day were 6.6 (SD = 0.9), 6.3 (SD = 1.3) and 6.6 (SD = 1.5) pre-, nine months and 48 months post-surgery, respectively in women, and 6.1 (SD = 1.3), 5.7 (SD = 1.4) and 6.1 (SD = 1.3) pre-, nine months and 48 months post-surgery, respectively in children.

Accelerometer measurements of levels of PA and ST three months before and nine and 48 months after maternal RYGB displayed no significant differences in MVPA, LPA or ST, between any given measure point, in women (Table 2). On the contrary, children decreased time spent in MVPA with 13.2 min/day (*p* = 0.041), time spent in LPA with 82.2 min/day (*p* < 0.001) and increased time spent sedentary with 110.5 min/day (*p* < 0.001) from three months before to 48 months after maternal RYGB. Mean cpm/day and LPA decreased significantly (*p* = 0.002 and *p* < 0.001, respectively) and ST increased (*p* < 0.001), from nine to 48 months after maternal RYGB. MVPA showed a non-statistically significant decrease from pre-surgery and nine months post-surgery (diff = −3.5 *p* = 0.557) and from nine months and 48 months post-surgery (diff = −9.7 *p* = 0.064). On average, women spent 3.8, 4.0 and 3.6%, whereas children spent 8.7, 8.2 and 7.0% of the day in MVPA, three months before and nine and 48 months after RYGB.

**Fig. 1:**
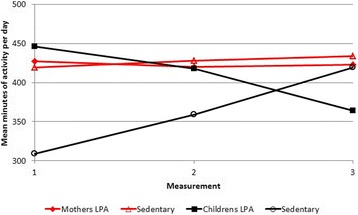
Means of light physical activity and sedentary time, in children and women three months before and nine and 48 months after maternal Roux-en-Y Gastric Bypass surgery

**Fig. 2:**
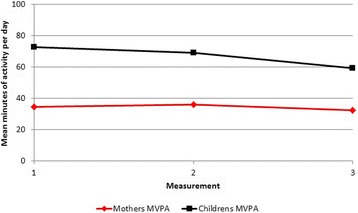
Means of moderate to vigorous physical activity, in children and women three months before and nine and 48 months after maternal Roux-en-Y Gastric Bypass surgery

Fifteen women (50%) increased, whereas 15 women (50%) decreased their time spent in MVPA from three months before to 48 months after RYGB. In addition, 16 women (53%) increased, whereas the reaming 14 women (47%) decreased their time spent sedentary from three months before to 48 months after RYGB. Twenty-three (58%), 36 (90%) and six (15%) children decreased time spent in MVPA, LPA and ST, from three months before to nine and 48 months after maternal RYGB, respectively. Means of daily minutes spent in MVPA, LPA and ST three months before and nine and 48 months after maternal RYGB in women and children are shown graphically in Figs. 1 and 2.

### Meeting the physical activity guidelines

**Fig. 3:**
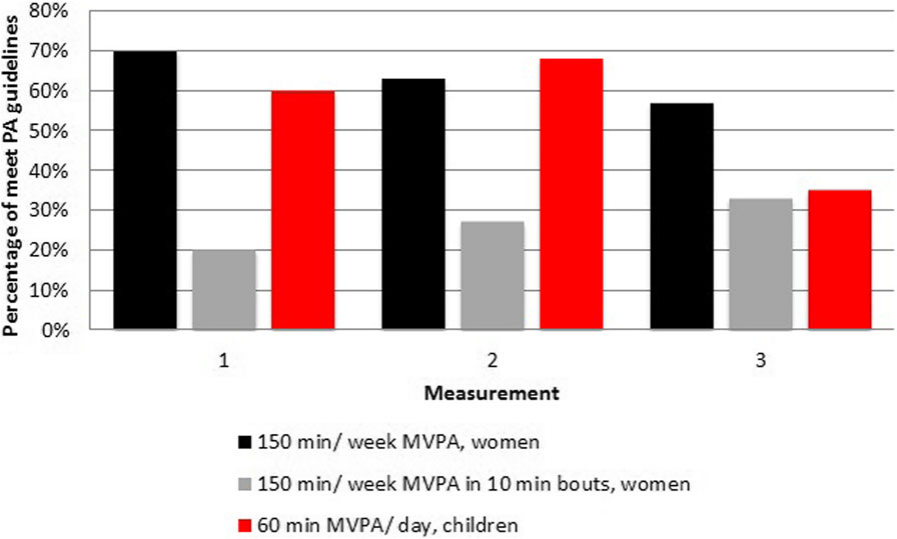
Percentage of children and women who meet current physical activity guidelines three months before and nine and 48 months after maternal Roux-en-Y Gastric Bypass surgery

Twenty percent three months pre-surgery, 27% nine and 33% 48 months post-surgery of women meet the current PA guidelines of at least 150 min of MVPA in 10-min bouts per week as recommended for the general adult population [8] (Fig. 3). During the same time frame, 70, 63 and 57% of the women reached the PA guidelines when total time spent in MVPA was analyzed. Children five to 17 years of age are recommended to engage in at least 60 min of MVPA each day [19]. This PA guideline is met by 60, 68 and 35% of children at three months before and nine and 48 months after maternal RYGB, respectively (Fig. 3). As shown in Tables 2 and 3 there were great variations between individuals at three months before and nine and 48 months after maternal RYGB in levels of PA and ST, and furthermore in their differences over time.

## Discussion

The present study assessed longitudinal changes in levels of PA and ST among 30 women undergoing RYGB and 40 appurtenant children from three months before through nine and 48 months after maternal surgery. Findings show that women undergoing RYGB do not change levels of PA or ST noteworthy, whereas appurtenant children significantly reduce levels of PA and increase ST from three months before to 48 months after maternal surgery. In addition, there is a trend towards progressively decreased levels of PA and increased ST in children from nine to 48 months after maternal RYGB. Furthermore, the prevalence of women undergoing RYGB meeting the PA guidelines is low three months before and nine and 48 months after maternal surgery, while 60% of appurtenant children met the PA guidelines before maternal RYGB and that number dropped to 35% after 48 months.

The present study presents, to our knowledge, the lengthiest follow-up time with objectively accelerometer measured PA and ST in women undergoing RYGB and appurtenant children. To date, objectively measured PA data is available up to three years after bariatric surgery [20]. However, those data on PA relies on indirect measures of MVPA and ST derived from pedometers measuring step counts in one minute bouts. Research has shown poor correlation between pedometer and accelerometer assessed PA in a free-living condition in obese women [21]. Hence, comparability between the present study and previous research with a two-year follow-up is somewhat limited. Previous research with one-year follow-up indicates that self-reported PA increases and time spent sedentary decreases among all family members when an adult family member undergoes bariatric surgery [9]. Contrary, objectively measured PA and ST in women undergoing RYGB do not increase substantially, with a reduction in children’s levels of MVPA (−11 min/day) and increases in ST (+ 53.7 min/day) nine months after maternal surgery [10]. This discrepancy between self-reported and objectively measured PA has previously been shown in individuals undergoing bariatric surgery, who report a greater disagreement between self-reported and objectively measured PA after compared with before surgery [22, 23].

One plausible explanation for the gradual decrease in PA and increase in ST among children, after maternal RYGB, may be attributed to the observed age dependent decrease in objectively measured PA, starting at five-years of age with a gradual decrease throughout childhood and adolescence [24].

Compared with previous studies assessing PA and ST in individuals undergoing bariatric surgery in the US, women in the present study spent more time in MVPA and less time sedentary both before and after surgery [14, 20]. Study population dissimilarities may account for these observed differences in levels of activity. For example, objective measures have shown that Swedish females on average engage in higher levels of MVPA compared with US females [25].

Twenty to 33% of women undergoing RYGB, in the present study, reached the recommended levels of PA [8] at all three measure points, whereas, more than half of the women accumulated more than 150 min of MVPA per week, at all three measure points, when total MVPA minutes, independent of bout length, were analyzed. However, the American College of Sports Medicine propose that at least 250 min of MVPA per week is required to prevent weight regain after substantial weight loss [26]. Twelve percent of women in the present study reach these levels of PA, at all three measure points. In addition, researchers have proposed that objectively assessed MVPA, reporting the sum of accumulated moderate to vigorous intensity PA as the primary feedback, needs to be five- to seven-fold greater than the 150 min per week target [27]. Only one woman in the present study, at all three measure points, reach these levels of PA when using this cut-off with at least 750 min of MVPA per week. The number of women meeting the recommended levels of PA, when analyzed as MVPA in 10 min bouts, increased somewhat from three months before to nine and then 48 months after RYGB. Conversely, total MVPA per week, independent of bout length, decreased progressively from three months before through nine and 48 months after RYGB. This may indicate that women to some extent engage in more structured PA, such as different forms of exercise, and less daily life MVPA, which has been show to decrease globally [28].

Major strengths of the present study lie in the longitudinal study design with three measure points over four years in both women undergoing RYGB and appurtenant children, and the objective measurements of PA, ST and anthropometrics that, by study design, enables control of effects that are constant (e.g. genetics) from before to after surgery. Anthropometrics, PA and ST were measured with standardized techniques and the same instruments at all three measure points. The Vm axis of the tri-axial accelerometer Actigraph GT3X+ used to measure levels of activity has been shown to accurately assess PA and ST in adults [11, 15] and children [16]. Moreover, both the accelerometer wear-time and data processing protocols followed best practice [29]. Although the accelerometer is unable to measure what types of activities are performed (e.g. bicycling and weight lifting), data has shown that activities are performed with similar occurrences before and after bariatric surgery [14].

Several limitations should be considered when interpreting the results. First, dissimilarities in hospital routines in identifying potential study participants made it impossible to gather information on individuals who declined to participate in the study. Second, participants were recruited from five hospitals with slightly different PA advices before and after surgery. Third, accelerometers may not fully capture all types movements, such as weight baring activities, thus, not provide an accurate assessment of total daily PA performed during free-living conditions [30]. Fourth, data on cut-offs developed using tri-axial Vm axis accelerometer data is sparse [11, 15]. Furthermore, accelerometer output from the Vm axis is not directly comparable with the more commonly used Vt axis from uni-axial accelerometers [31]. Hence, results from the present study assessing tri-axial Vm accelerometer data cannot be directly compared with data from older uni-axial accelerometers. Fifth, lack of statistical power due to few observations and large variation on several outcome variables makes the results sensitive to outliers. There is great individual variability in longitudinal changes of PA and ST among women undergoing RYGB and appurtenant children through three months before to nine and 48 months after maternal surgery. Thus, results from the present study on mean average differences in PA and ST across all three measure points should be interpreted with caution. Sixth, the present study did not have a control group, therefore the presented data is restricted to be descriptive. A control group would though have been hard to compare to since individuals with obesity that does not perform bariatric surgery and loose similar amounts of weight might differ substantially from our population. Seventh, the low prevalence of type 2 diabetes in our sample implies a healthier population compared to the typical RYGB patient. Last, the sample size in the present study is small; however, studies with similar design have used comparable or even smaller study populations and shorter follow-up time [9, 22].

## Conclusions

In conclusion, the present study shows no significant longitudinal differences in objectively measured PA and ST in women undergoing RYGB from three months before to nine and 48 months after surgery. However, during the same time frame, levels of PA decreased and ST increased significantly among appurtenant children. The prevalence of women meeting the current PA guidelines are low before maternal RYGB and remain low nine and 48 months after surgery, while the prevalence of appurtenant children that meet the recommended guidelines decrease substantially from before to 48 months after maternal RYGB. This implies the need to incorporate effective pre- and post-surgery PA counselling specific to this population.

## Abbreviations

BMI: Body mass index
LPA: Light physical activity
MVPA: Moderate to vigorous physical activity
PA: Physical activity
RYGB: Roux-en-y gastric bypass
ST: Sedentary time
